# Solitary central osteoma of mandible in a geriatric patient: Report and review

**DOI:** 10.4317/jced.52792

**Published:** 2016-04-01

**Authors:** Kumar Nilesh, Ravi B. Bhujbal, Ajay G. Nayak

**Affiliations:** 1 Associate Professor, Department of Oral & Maxillofacial Surgery, School of Dental Sciences, KIMSDU, Karad, Maharashtra, India; 2 Senior Lecturer, Department of Oral & Maxillofacial Surgery, Yogita Dental College and Hospital, Ratnagiri, Maharashtra, India; 3 Associate Professor & H.O.D, Department of Oral Medicine & Radiology, School of Dental Sciences, KIMSDU, Karad, Maharashtra, India

## Abstract

Solitary central osteomas of jaw are extremely rare lesions with only few previously documented cases. This paper reports a case of large solitary central osteoma involving mandible symphysis- parasymphysis region in an elderly female patient. A brief review of similar cases reported in the literature is also provided in this paper.

** Key words:**Osteomas, osteogenic,bone, tumor, jaw, mandible.

## Introduction

Osteomas are rare benign lesions characterized by proliferation of compact and/or cancellous bone. Three variants of ostemoa have been described in literature - peripheral (periosteal), central (endosteal), and extra-skeletal. The peripheral osteoma arises from periosteum and presents as peripheral mass attached to the cortical plate, whereas the central variants are extremely rare and arises within the bone, developing from endosteum. Extra-skeletal soft tissue osteomas are usually seen within muscles (1).

Multiple osteomas may present as part of Gardner’s syndrome, an autosomal dominant disease characterized by gastrointestinal polyps, soft tissue tumors, and multiple impacted or supernumerary teeth. Solitary osteomas in oral and maxillofacial region, often involve the skull bone, the frontal bone (sinus) being the site of predilection (2). Solitary central osteoma (SCO) involving the jaw bones is a very rare occurrence with only twelve cases reported in English literature since 1955 (3). Out of the 12 cases, eight were seen in mandible, with common site of occurrence in premolar-molar region (4). In view of the rarity of this pathology, this paper reports a case of large SCO of the mandible symphysis-parasymphysis region in an old female patient.

## Case Report

A 75 year old female reported to our clinic with complaint of painless intraoral swelling over the lower anterior jaw, causing difficulty in speech and interference in tongue movements. The patient had first noticed the swelling five years earlier. No history of facial trauma was reported. The patient was under medication for hypertension for the past fifteen years. She gave a history of cardiac surgery five years back and was subsequently on anti-platelet therapy. History of multiple teeth extractions over a period of time was given, of which no record was available. On examination a non-tender bony hard swelling was present over mandible alveolus, extending across the midline from 32 to 44 region, measuring about 5 cm x 3 cm. The swelling extended lingually to the tip of the tongue. The mucosa overlying the swelling was normal. Patient was partially edentulous with poor oral hygiene. Mandibular left canine and premolar teeth present over lateral aspect of the swelling exhibited grade III mobility. Other mandibular anterior teeth were missing.

**Figure 1:**
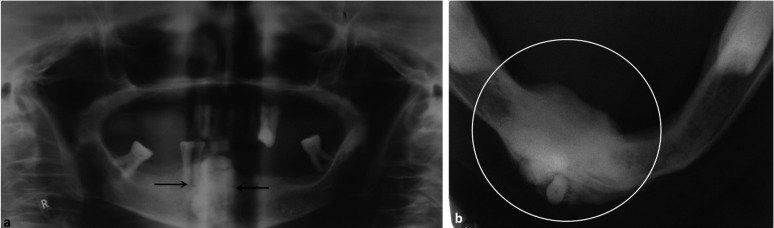
Orthopantomogram (a) showing radiopaque lesion in anterior mandible; mandibular occlusal view (b) showing the size and extent of lesion.

**Figure 2:**
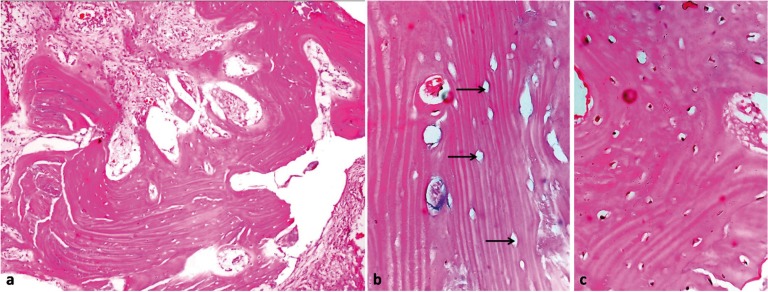
H & E Section a) showing compact bone with fibro-fatty marrow tissue (10 X magnification); b) section showing sclerotic bone with empty lacunae (40 X magnification); c) section showing compact bone with osteocytes (40 X magnification).

Orthopantamogram (OPG) showed a radiopaque lesion in mandible anterior region, extending from the crest of the alveolar ridge upto the inferior border of the mandible, superoinferiorly and measuring about 5 cm in its greatest dimension. The lesion had well defined margins and could be demarcated from the surrounding normal bone. A mandibular occlusal radiograph showed radiopaque mass expanding the buccal and lingual cortices (Fig. 1). Based on clinical and radiological examination a working diagnosis of solitary central osteoma of mandible was made. An incisional biopsy was planned and executed under local anesthesia. Microscopy of the decalcified sections of the specimen (Fig. 2) revealed areas of dense compact bone with osteocytes. Large amount of areas showed homogeneous mass of bone which appeared sclerotic with absence of osteocytes and osteblasts. Fibro-fatty marrow was seen with a moderate inflammatory infiltrate. The above features confirmed diagnosis of osteoma.

**Figure 3:**
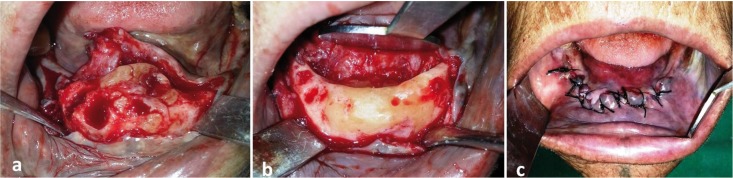
Intraoperative picture showing a) exposed lesion; b) recontoured mandible and c) surgical site after closure.

**Figure 4:**
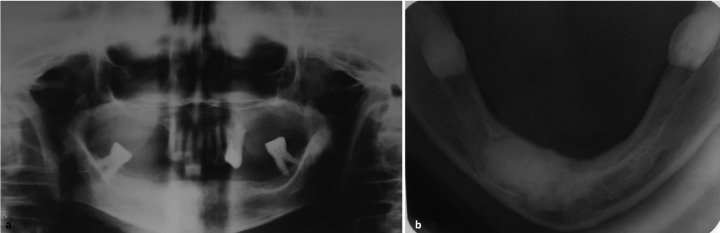
One year postoperative Orthopantomogram a) and b) mandibular occlusal view.

For management of the lesion, both option of complete excision under general anesthesia and a conservative approach by recontouring of the jaw under local anesthesia was discussed with the patient, physician and anesthesiologist. Considering her age, medically compromised status and non-malignant, non-infiltrative nature of the lesion conservative surgical management was preferred choice after consensus. After attaining adequate regional anesthesia, a crestal incision was place over the alveolar bone and envelop flap raised, exposing the bony mass. The lesion appeared more yellowish and could be easily differentiated from the surrounding normal bone. The mandibular right canine and first premolar were extracted. The excess hard tissue overlying the alveolar ridge was contoured. The flap was approximated and sutured (Fig. 3). The excised specimen was submitted for histopathological evaluation, which showed a hard mass consisting entirely of dense lameller compact bone. The clinical, radiographical and microscopic findings confirmed diagnosis of central osteoma of mandible. The post-operative period was uneventful and the healing was within the anticipated time period. The patient was followed up after one year, where satisfactory bony contour of anterior mandible, with no further growth of the residual lesion was noted (Fig. 4). The patient was advised long term follow-up to rule out any recurrence in future.

## Discussion

Osteomas are rare benign osteogenic tumors, characterized by the proliferation of either or both compact and cancellous bone. The reported age at onset ranges from 14 to 58 years, with a mean age of 29 years. Males are affected more frequently than females, in a ratio of approximately 2:1 (2). They are almost exclusively seen in craniofacial skeleton, of which frontal and paranasal sinuses are more commonly involved sites. Multiple osteomas of the jaw area usual feature of Gardner syndrome, whereas nonsyndromic cases are uncommon and presents typically as a solitary lesion. They can develop as peripheral masses attached to the cortical plates or as central lesions arising from endosteal bone. Larrea-Oyarbide *et al.* (5) in one of the largest retrospective review studied 106 patients diagnosed with 132 osteomas of the craniomaxilofacial region. 49% of the patients had peripheral osteomas whereas 29% were of central type. A large number of cases (21%) were seen in paranasal sinuses. Kaplan *et al.* in 2008 (6) identified 91 well documented cases of solitary osteoma of the jaws. It was found that 93.4% of the cases were peripheral,with only 6.6% comprising central osteomas (ratio of 14:1 in favor of peripheral osteomas). Only eight cases of SCO involving the mandible have been reported since 1955. (6-11). Seven of these cases were seen in premolar-molar region, while one presented in canine-premolar region. In this present case the lesion was seen in a 75 years old female and was located over the mandible symphysis-parasymphysis region across the midline, a feature that has not been reported before.

Although various hypotheses have been proposed, the etiology of osteoma is still unclear and hasn’t been substantiated as yet. Some researchers believe osteomas to be reactive lesions, rather than neoplasms, representing the end stage of an injury and/or inflammation (4). The lesion in our patient was in close proximity of mandibular anterior teeth with chronic periodontitis. Chronic infection along with multiple tooth extraction in the region could possibly have triggered the osteoproliferative process, giving rise to the lesion.

SCO of mandible has been reported in both genders (male- 3 cases, female-5 cases); with an age range of 13-67 years ( Table 1). Smaller sized SCO is usually asymptomatic and get discovered during routine radiographic examination. Larger lesion invariably present as bony hard swelling. In 50% (4 patients) of previously reported cases, swelling was associated with pain. Although nerve involvement is not a feature of central osteoma, Fritz *et al.* reported a case SCO causing lower lip paresthesia due to direct nerve compression (9). On radiography, SCO usually appears as radiopaque lesion with well-defined margin, delineating it from the surrounding jaw bone. One previous reported case (8) presented as mixed lesion, while two cases (7,10) had irregular margins on radiographs. SCO should be differentiated from other radiopaque lesions of the jaw, including central ossifying fibroma, conden-sing osteitis, idiopathic osteosclerosis, osteoblastoma, cementoblastoma, and complex odontoma. The reported lesion manifested as radiopaque mass with defined margin along with expansion of the bone, which was sufficient to support the diagnosis of central osteoma. The clinical diagnosis was confirmed on histological evaluation.

**Table 1:**
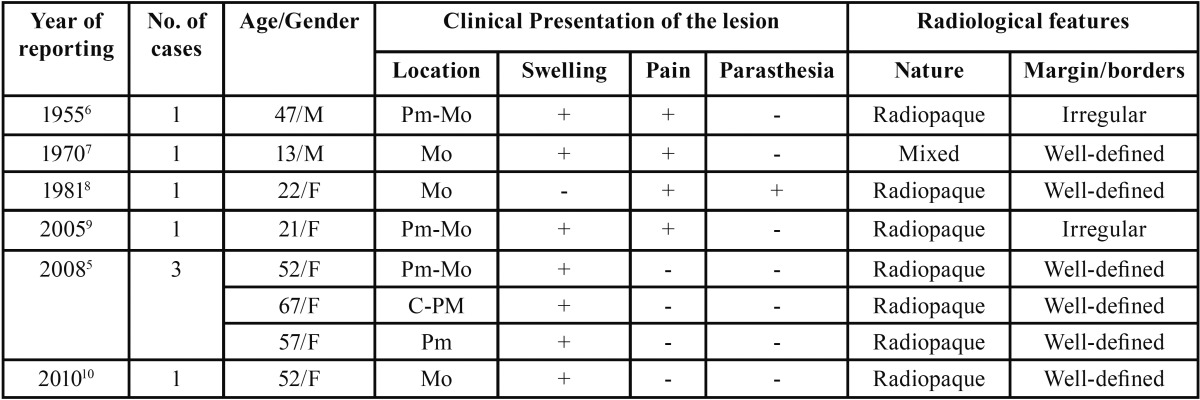
Review of previously reported cases of SCO of mandible.

Management of osteoma depends on its presentation. Smaller asymptomatic SCO generally does not require any treatment. Surgical intervention is indicated for lesions which are large, symptomatic, and painful or causes functional impairment. Excision is usually simple in pedinculated peripheral osteomas. Larger central variants require wider area of bone resection, thus increasing the surgical risks and postoperative complications. Recurrence is unlikely. In the present case lesion was managed conservatively by surgical recontouring of the anterior mandible with functionally and aesthetically favorable outcome.

Due to the rarity of reported cases in literature, this paper puts forth the ninth case of SCO of mandible. Unlike the other eight cases our case presented in geriatric patient and was located in anterior mandible crossing the midline.
